# A Bayesian decision support tool for efficient dose individualization of warfarin in adults and children

**DOI:** 10.1186/s12911-014-0128-0

**Published:** 2015-02-07

**Authors:** Anna-Karin Hamberg, Jacob Hellman, Jonny Dahlberg, E Niclas Jonsson, Mia Wadelius

**Affiliations:** Department of Medical Sciences, Clinical Pharmacology and Science for Life Laboratory, Uppsala University, SE-751 85 Uppsala, Sweden; Department of Engineering Sciences, Uppsala University, Box 256, SE-751 05 Uppsala, Sweden; Pharmetheus AB, Dag Hammarskjölds väg 52B, SE-752 37 Uppsala, Sweden

**Keywords:** Anticoagulation, Bayesian forecasting, Dose individualization, Population PK/PD-models, Warfarin

## Abstract

**Background:**

Warfarin is the most widely prescribed anticoagulant for the prevention and treatment of thromboembolic events. Although highly effective, the use of warfarin is limited by a narrow therapeutic range combined with a more than ten-fold difference in the dose required for adequate anticoagulation in adults. An optimal dose that leads to a favourable balance between the wanted antithrombotic effect and the risk of bleeding as measured by the prothrombin time International Normalised Ratio (INR) must be found for each patient. A model describing the time-course of the INR response can be used to aid dose selection before starting therapy (*a priori* dose prediction) and after therapy has been initiated (*a posteriori* dose revision).

**Results:**

In this paper we describe a warfarin decision support tool. It was transferred from a population PKPD-model for warfarin developed in NONMEM to a platform independent tool written in Java. The tool proved capable of solving a system of differential equations that represent the pharmacokinetics and pharmacodynamics of warfarin with a performance comparable to NONMEM. To estimate an *a priori* dose the user enters information on body weight, age, baseline and target INR, and optionally *CYP2C9* and *VKORC1* genotype. By adding information about previous doses and INR observations, the tool will suggest a new dose *a posteriori* through Bayesian forecasting. Results are displayed as the predicted dose per day and per week, and graphically as the predicted INR curve. The tool can also be used to predict INR following any given dose regimen, e.g. a fixed or an individualized loading-dose regimen.

**Conclusions:**

We believe that this type of mechanism-based decision support tool could be useful for initiating and maintaining warfarin therapy in the clinic. It will ensure more consistent dose adjustment practices between prescribers, and provide efficient and truly individualized warfarin dosing in both children and adults.

**Electronic supplementary material:**

The online version of this article (doi:10.1186/s12911-014-0128-0) contains supplementary material, which is available to authorized users.

## Background

Warfarin is one of the most commonly prescribed anticoagulants in both adults and children [1], with over 33 million prescriptions in 2011 [2]. In spite of the recent introduction of the new oral anticoagulants (NOACs), i.e. dabigatran, rivaroxaban and apixaban, warfarin still remains the most prescribed anticoagulant with 90% of Swedish patients receiving warfarin and only 10% receiving a NOAC during 2013 [3]. Although it has been in clinical use for over 50 years, warfarin therapy is still challenging due to a narrow therapeutic range and considerable variability in response to a given dose. Known contributing factors to the between- and within-subject variability among adult patients include, age, concurrent medications and/or health conditions, vitamin K intake and genetic polymorphisms in two genes, *CYP2C9* and *VKORC1* [4-6]. In a systematic review and meta-analysis, patients with atrial fibrillation (AF) receiving warfarin spent 61% of the time within, 13% above, and 26% below the target INR of 2-3 [7]. In a US study that was published in 2011, the frequency of warfarin-induced bleeding was reported to be 15% to 20% per year, with life-threatening or fatal bleeding rates as high as 1% to 3% per year [8]. Annual total health care costs were estimated to be 65% and 49% higher for AF patients with a warfarin-induced intracranial hemorrhage or a major gastrointestinal bleeding, respectively, than the costs for patients with no bleeding events [9].

Dose individualization to minimize the risk for over- or under-dosing can be made i) before starting therapy (*a priori*) and/or ii) after therapy has been initiated (*a posteriori*) and may range in complexity from body size based dosing to utilization of advanced mechanism based mathematical and statistical models. There are several published pharmacogenetic prediction models for *a priori* dose individualization of warfarin for both adults [4,5,10] and children [11-13]. These dosing algorithms aim to predict the expected maintenance dose. A more refined way to achieve individualized dosing is to combine methods for *a priori* individualization with methods for *a posteriori* dose revisions, using a Bayesian approach [14,15]. The latter utilizes knowledge of the population distribution of the model parameters for the drug. The most likely parameters for an individual can be obtained using measurements of drug concentrations [16,17] or drug responses [18,19]. These parameters can be used to calculate the dose that most probably results in the target response in that particular individual. By using a predictive model combined with Bayesian forecasting, warfarin dosing can be truly personalized, resulting in rapid achievement of therapeutic anticoagulation without increasing the risk of over-anticoagulation.

**Figure 1:**
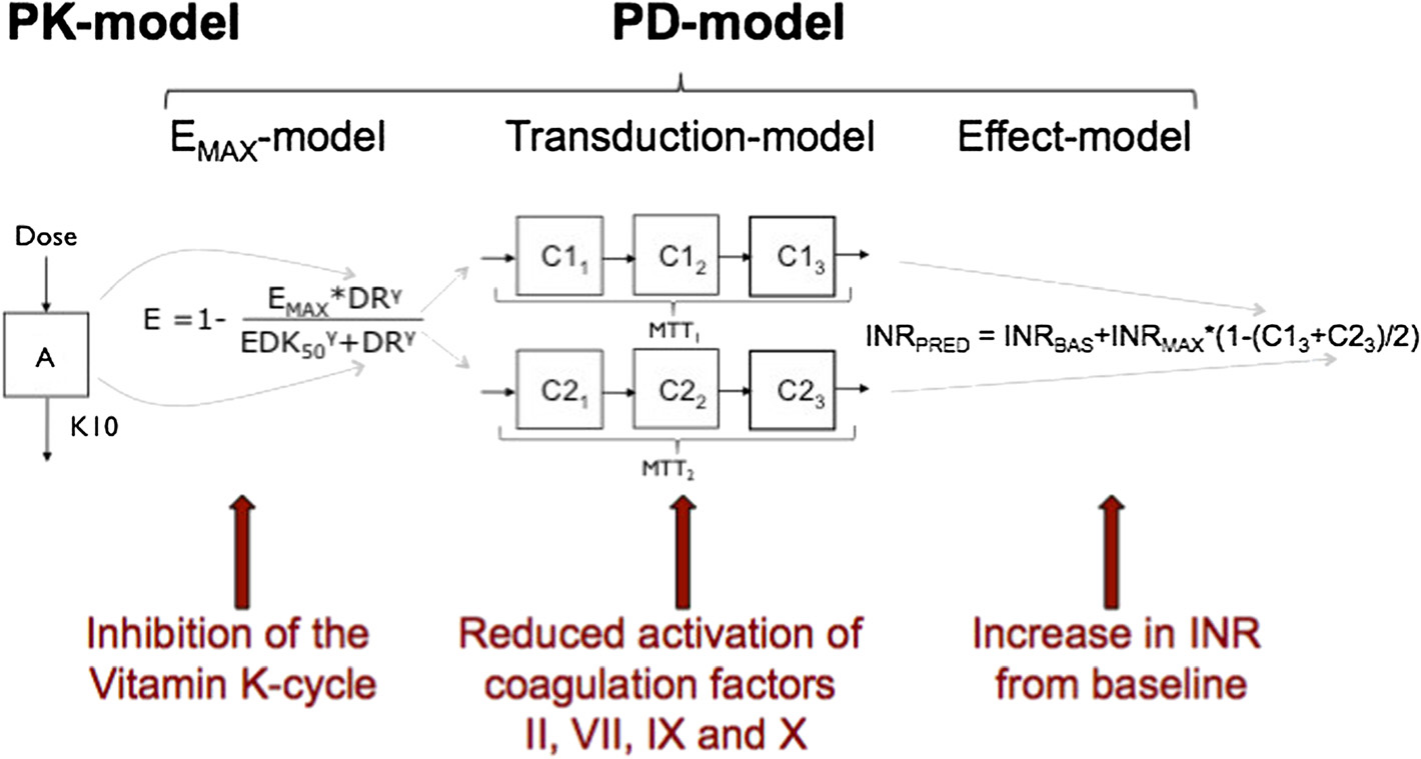
**Schematic picture of PKPD**-**based warfarin model.** This is a schematic picture of the basic structure of the published PKPD-model for warfarin. The predictors necessary for individual dose predictions (e.g. *CYP2C9* and *VKORC1* genotype, age and bodyweight, baseline and target INR) are not included in the picture.

In this paper we present a warfarin dose decision tool (available as Additional file 1) developed from a published population model for warfarin. The model is founded on pharmacokinetic (PK) and pharmacodynamic (PD) principles [20-22] and is schematically presented in Figure 1. The tool can be used *a priori* to predict the most probable dose to reach a given target INR, or to predict the most probable INR response to a given dose. It can also be used *a posteriori* to guide dose revisions using a Bayesian forecasting method. The model was developed on longitudinal data from more than 1,500 warfarin treated adults [20-21], and then bridged theoretically to children 0.18 years old [22]. There is a time delay between warfarin dosing and INR response, and this is captured in the model by inclusion of a transduction model, consisting of two parallel compartment chains, where n is the number of compartments in each chain and MTT is the mean transit time through each chain. Two parallel chains were necessary to describe the exposure–response relationship over time, and is possibly a reflection of differences in half-lives of the coagulation factors affected by warfarin and that influences the INR response [20]. The general form of the model is given by the following set of equations:

1 $$ \frac{dA}{dt} = -{k}_e*A $$

2 $$ DR={k}_e*A $$

3 $$ EFF = \frac{{E_{MAX}}^{\gamma }*D{R}^{\gamma }}{ED{K_{50}}^{\gamma }+D{R}^{\gamma }} $$

4 $$ \frac{dC}{dt}=\frac{\left(1- EFF\right)*n}{MTT}-\frac{C*n}{MTT} $$

A represents the amount of drug in the body at any time after one or more administrated doses. The first-order elimination rate constant, k_e_ (derived from the ratio of the PK parameters clearance and volume of distribution, k_e_ = CL/V), governs the level of drug amount A at any given time and also the distribution of the drug to the site of action (Equation 1). The dose rate DR (Equation 2) together with one of the PD sub models (Equation 3), defined by the parameters E_max_ (the maximum degree of inhibition which is set to 1) and EDK_50_ (the dose rate resulting in 50% of maximum inhibition), determines the extent of inhibition of the vitamin K cycle and the inhibition of coagulation factor activity. dC/dt (Equation 4) describes the fraction of activated coagulation factors remaining at any given time. The initial conditions of dA/dt and dC/dt are set to 0 and 1, respectively, i.e. no drug in the body and 100% activity of coagulation factors before start of therapy. The INR at any given time is predicted by the following equation:

5 $$ IN{R}_{PRED}= IN{R}_{BASE}+ IN{R}_{MAX}*\left(1-\left(C{1}_3+C{2}_3\right)/2\right) $$

*INR*_*BASE*_ represents the INR at baseline (before warfarin treatment), *INR*_*MAX*_ is a theoretical maximal increase from baseline INR (fixed to 20 as in [21]), and *C1*_*3*_ and *C2*_*3*_ represents the coagulation factor activity in the terminal compartment in each transit chain. Each transit chain is defined by a set of differential equations as exemplified below for the first chain C1:

6 $$ \frac{dC{1}_1}{dt}=\left(1- EFF\right)*\frac{3}{MT{T}_1}-C{1}_1*\frac{3}{MT{T}_1} $$

7 $$ \frac{dC{1}_2}{dt}=C{1}_1*\frac{3}{MT{T}_1}-C{1}_2*\frac{3}{MT{T}_1} $$

8 $$ \frac{dC{1}_3}{dt}=C{1}_2*\frac{3}{MT{T}_1}-C{1}_3*\frac{3}{MT{T}_1} $$

A complete description of the underlying warfarin model can be found in the papers describing the model development in NONMEM [20-22]. NONMEM is the most commonly used software for non-linear mixed effects modeling of PK and PD data [23].

## Implementation

### Tool development

One of the published warfarin models [22] was transferred from NONMEM to a new graphical user interface built with Java Swing components using NetBeans [24]. NetBeans refers both to a platform framework for Java applications, and to an open source integrated development environment, supporting development of all types of Java applications. The differential equations in the Java application are solved using Heun’s method, a second-order Runge-Kutta method, which is a numerical procedure for solving ordinary differential equations that is both fast and easy to implement using vectors. Heun’s method is also stable for this type of differential equations and has a high numerical precision. The end result is a Java application that, for a subject with a given set of covariates, can estimate the maintenance dose for a pre-specified target INR or predict the INR response for a pre-specified dose regimen. There are two main windows in the application, one for *a priori* predictions and one for *a posteriori* predictions. The rate constant k_e_ is referred to as k10 in the tool.

#### A priori predictions

The tool needs input data regarding the patient in order to operate. Data on age, weight, *CYP2C9* and *VKORC1* genotype, baseline INR and target INR range are required both for dose estimation and for INR prediction. If genotype information is missing, the tool will use the most common genotype combination, conditioned on ethnicity [25,26]. This means that for *CYP2C9* all subjects with missing genotype information will be coded as *1/*1 i.e. the genotype with the highest dose requirement. For *VKORC1* the tool will use A/G for Caucasians (intermediate dose requirement), A/A for Asians (low dose requirement) and G/G for Africans (high dose requirement). If baseline INR is missing the tool will use a default value of 1. The dosing interval has a default value of 24 hours, i.e. one dose per day, but this can be changed manually if another dosing interval is preferred. Common to all *a priori* predictions is that the model will use the typical (mean) parameter estimates conditioned on the patient’s age, bodyweight and *CYP2C9* and *VKORC1* genotype.

#### Estimation of dose

**Figure 2:**
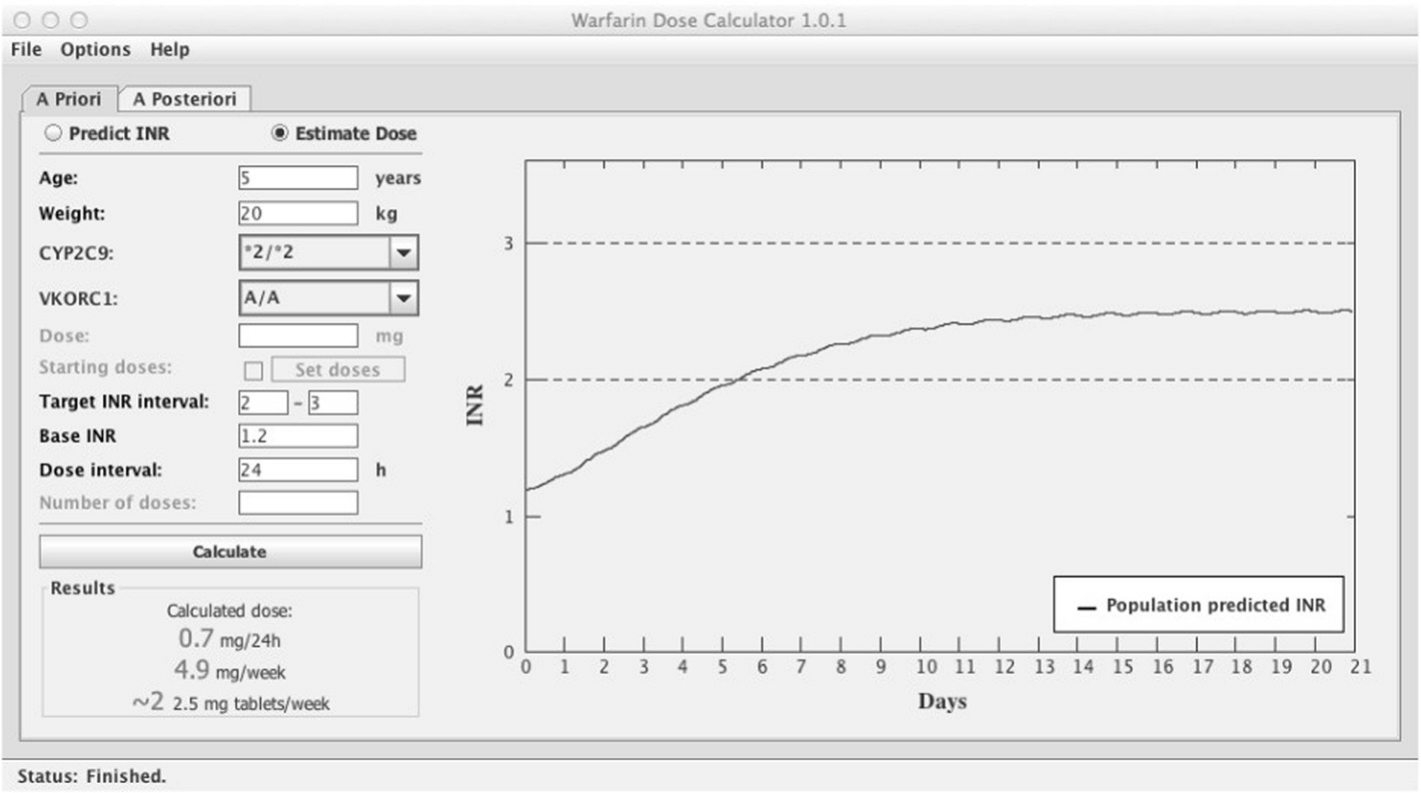
**Example of the** ***a priori*** **dose estimation function.** This shows an example of an *a priori* dose estimation for a 5 year old child, with bodyweight 20 kg, genotypes *CYP2C9* *2/*2 and *VKORC1* A/A, with a target INR of 2.0-3.0 and a baseline INR of 1.2. The estimated maintenance dose is 0.7 mg/24h, or 4.9 mg/week. The graph indicate that with this dose regimen, time to reach a target INR is ~6 days, and time to steady state is ~12 days.

To calculate the dose most likely to achieve the target INR, the option “Estimate dose” is chosen; see example in Figure 2. The tool uses the mean of the specified target INR interval as the target INR for which a dose should be estimated. The tool starts with a daily dose of 10 mg and calculates the expected INR after 100 daily administrations of the same dose, to ascertain that steady state conditions are reached. Depending on if the calculated INR is lower or higher than the target INR, the dose will be adjusted automatically in an iterative process until the calculated mean INR at steady state equals the target INR (Target INR ± 1%). The criteria for steady-state is met when the change in INR between two doses does not exceed 1%. To illustrate the expected time course to a therapeutic and stable INR, the output is presented as a plot of the predicted typical INR curve from the 1^st^ dose until steady state is reached. In addition, a text field shows the predicted maintenance dose in mg/day, mg/week and the number of 2.5 mg tablets per week that is closest to the estimated weekly dose. The latter is an adaptation to Swedish conditions where only a 2.5 mg tablet strength is marketed. The target INR range is marked in the plot to support the interpretation of the predicted INR curve.

#### Prediction of INR

**Figure 3:**
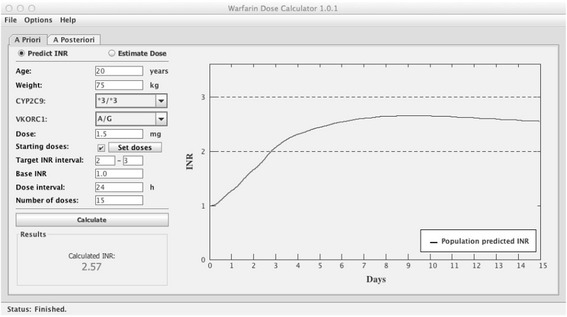
**Example of the** ***a priori*** **INR prediction function.** This shows an example of an *a priori* INR prediction for a 20 year old, with bodyweight 75 kg, genotypes *CYP2C9* *3/*3 and *VKORC1* A/G, with a target INR of 2.0-3.0 and a baseline INR of 1. The predicted INR after a total of 15 doses, including a 3-day loading dose regimen of 7.5 mg, 5 mg and 5 mg (not seen here but defined in the Set doses option) and followed by daily doses of 1.5 mg, is an INR of 2.57. The graph indicate that a target INR is reached after ~3 days with this dose regimen.

When the tool is used to predict an INR (See example in Figure 3), the user has to specify the dose and the number of days this dose should be repeated. The output is presented both as a plot of the predicted INR curve for the number of days specified, and as a text field showing the predicted INR at the end of this period. If steady state conditions have been reached the tool will display the mean INR over a dosing interval. If steady state conditions are not yet reached, the presented value is the predicted INR at 16 hours after last dose. The time point was chosen to reflect the clinical situation in Sweden, where INR is commonly monitored in the morning approximately 16 hours after last dose. There is also an option to predict INR after administration of a loading dose regimen. To do this “Starting doses” must be ticked, and the “Set doses” window opened. The user can specify a number of individual doses by entering the dose per day. If individual doses are chosen for the first three doses (e.g. Dose 1: 7.5 mg, Dose 2: 5 mg, Dose 3: 5 mg) and the total number of doses for the INR prediction is set to 15, the tool will automatically use the dose specified in the main window for the remaining 12 doses. Figure 3 shows the output from the example above with a 3-day loading dose regimen followed by 1.5 mg per day on Day 4-15.

### A posteriori predictions

Once treatment has been initiated and one or more INR observations are available, the tool can be used to suggest a tailored maintenance dose based on individualized parameter estimates. This is done in several steps using a Bayesian approach. The first step is to estimate individual model parameters, and this is done using Powell’s method. The tool then uses the individual model parameters in the next step, which can be either dose estimation or INR prediction. As more observations become available, the individual model parameters become more refined and specific to the individual patient. This is expected to increase the accuracy and precision of the dose and INR predictions.

#### Estimation of individual model parameters

**Figure 4:**
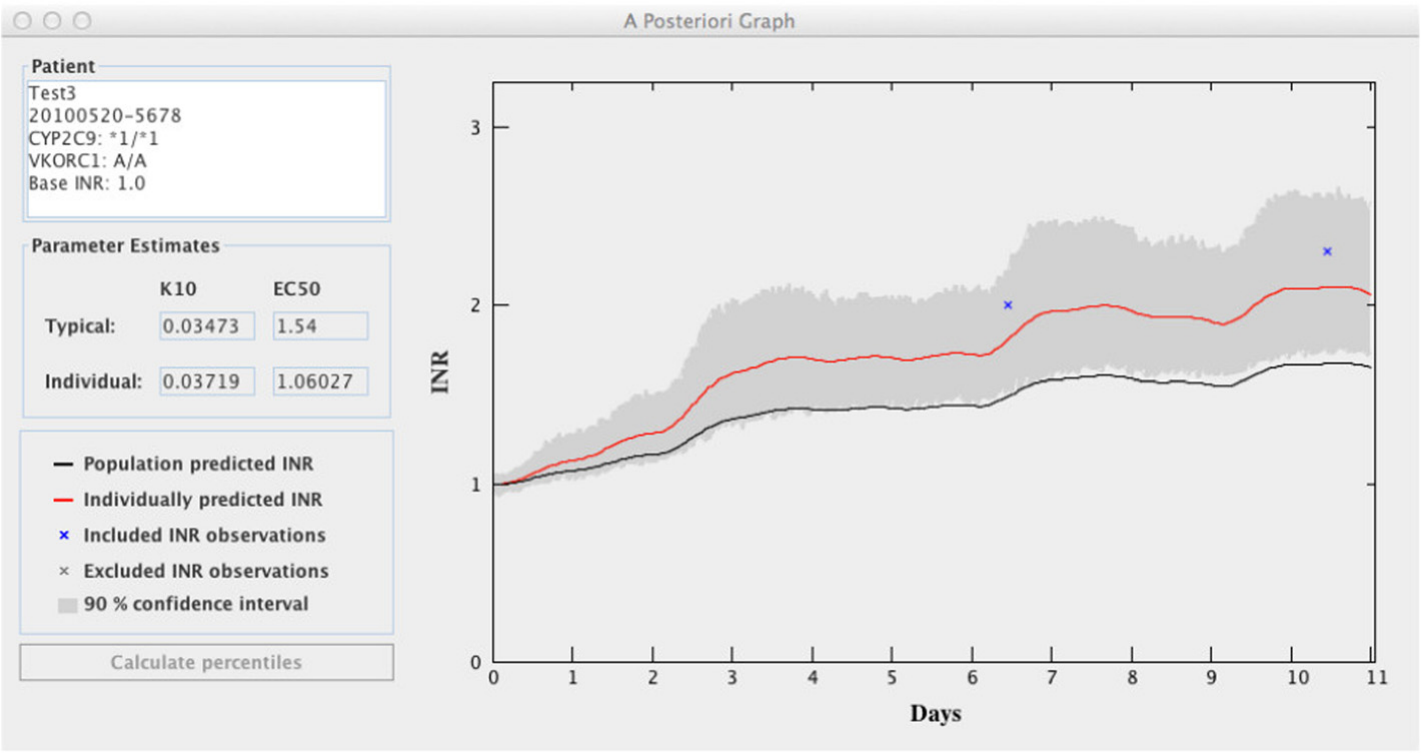
**Example of the estimation of individual parameters.** This provides an example of the output from the estimation of individual model parameters, showing both typical and individual parameter estimates, and the population predicted (black) and the individually predicted (red) INR curves for a given dose history. The individually predicted INR is presented with an optional 90% confidence interval.

For estimation of individual model parameters the tool requires patient specific information on demographics, initial warfarin doses and INR observations, including time of dosing and blood sampling for INR. The information can be entered either manually, or be imported from an Excel-file (see Additional files 2 and 3 for details on naming of files and required data format). When the data have been entered, click on “Estimate” to get the individual model parameter values for k10 and EC_50_. The output is presented in a new screen (see Figure 4) as a text field showing the typical (mean) parameter estimates for k10 and EC_50_ and the individual parameter estimates, and as a plot of the population predicted INR curve (in black) and the individually predicted INR curve (in red). The patient’s INR observations are also shown in the plot, which gives the user a chance to evaluate the individual fit. Optionally the individually predicted INR curve can be presented with a 90% confidence interval, to include uncertainty in the individual parameter estimates and the residual variability due to e.g. random errors in delivered dose, blood sampling time and/or INR measurements. When the individual model parameters have been estimated, select “Estimate Dose/INR” to get a new screen with the options “Estimate dose” and “Predict INR”.

#### Estimation of dose

**Figure 5:**
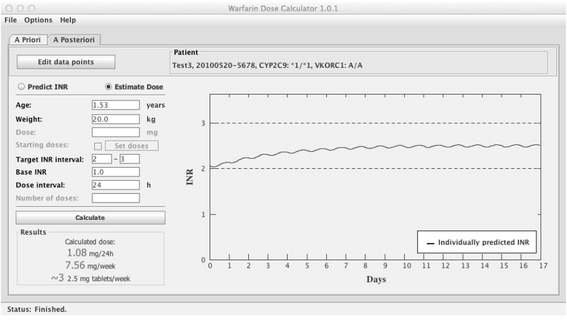
**Example of the** ***a posteriori*** **dose estimation function.** This shows an example of an *a posteriori* dose estimation for a 1.53 year old child, with bodyweight 20 kg, genotypes *CYP2C9* *1/*1 and *VKORC1* A/A, and target INR 2.0-3.0 and a baseline INR of 1 using the individual model parameters estimated in Figure 4. The estimated *a posteriori* dose is 1.08 mg/24 h, or 7.56 mg/week. The graph shows the predicted INR curve after administration of the estimated daily dose (1.08 mg).

Figure 5 shows a screen shot of the dose estimation option. The tool now uses the individual model parameters to suggest a tailored maintenance dose. The output is presented as a plot, with the individually predicted INR curve after administration of the tailored maintenance dose, starting from its current position (Day 0 in the plot). It is also presented as a text field showing the predicted dose in mg/day, mg/week and the corresponding number of 2.5 mg tablets per week. The target INR range will be displayed in the plot together with the individually predicted INR curve.

#### Prediction of INR

When predicting INRs, the user has to specify a dose and the number of days this dose should be repeated. The output is a plot of the predicted INR curve for the number of days specified, and a text field showing the predicted INR at the end of the treatment period. If steady state conditions have been reached the tool will display the mean INR over the dosing interval. If steady state conditions are not yet reached, the predicted INR at 16 hours after last dose is presented. This function may be useful e.g. in situations where it is not feasible to administer the same dose every day with available formulations. Thus, when different daily doses are required, the tool can visualize the variability in INR response that this regimen is expected to introduce. The tool can also be used to predict when warfarin should be discontinued to reach below a certain INR value at a given point in time, which can be of use e.g. before a planned surgical procedure.

## Results and discussion

**Figure 6:**
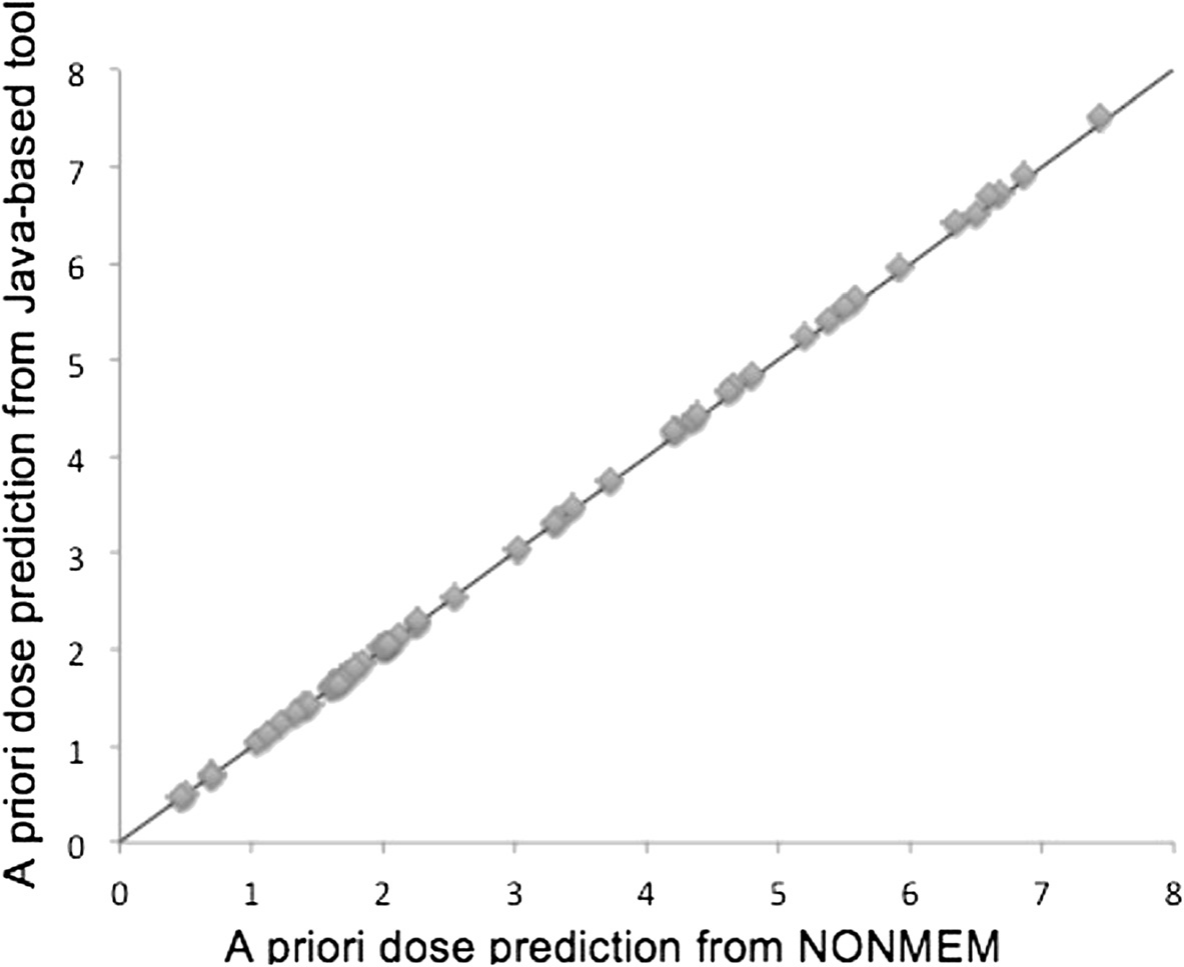
**Comparison of** ***a priori*** **dose predictions.** This figure provides results from a comparison of *a priori* dose predictions from NONMEM and the Java-based tool. The validation was performed using treatment data from 49 external children, and the results indicated no differences in computational performance between the two methods.

**Figure 7:**
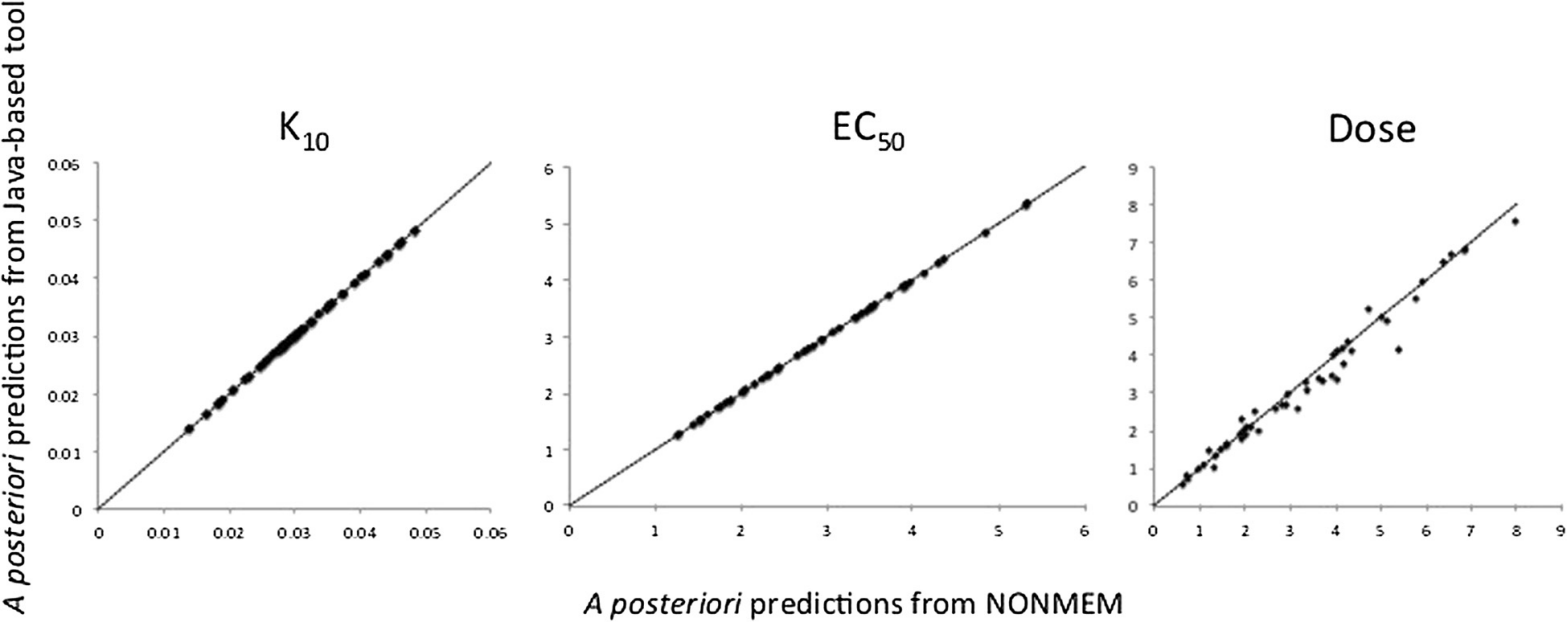
**Comparison of individual parameter estimates and** ***a posteriori*** **dose predictions.** This figure provides results from comparisons of individual parameter estimates (K_10_ and EC_50_) and *a posteriori* dose predictions from NONMEM and the Java-based tool. The validation was performed using treatment data from 49 external children, and the results indicated minor differences in computational performance between the two methods.

The computational performance of the Java-based tool was evaluated by comparing the output with the POSTHOC function in NONMEM version 7 as the reference. This was done using treatment data (one to three INR observations) from a total of 49 children [22]. *A priori* predicted maintenance doses and empirical Bayes estimates of individual parameters and *a posteriori* predictions of maintenance doses from the tool and from NONMEM were compared. Results from *a priori* comparisons are presented in Figure 6, and from *a posteriori* comparisons in Figure 7. There were no differences in *a priori* maintenance dose predictions with the Java based tool compared to NONMEM, but a mean difference in *a posteriori* maintenance dose predictions of 5.0% (SD 6.7%). There was a systematic difference in *a posteriori* maintenance dose predictions, with a bias (mean prediction error, MPE) of -0.104 mg and an imprecision (relative mean prediction error, RMPE) of 0.192 mg. Performance was benchmarked on a MacBook Pro with a 3.06 GHz Intel Core 2 Duo processor. Run times for a typical *a priori* prediction was a few seconds. For *a posteriori* predictions, run times were correlated with the length of the treatment history used for computation of empirical Bayes estimates. However, total run times, including estimation of a tailored maintenance dose, seldom exceeded 1 minute. A typical run time for *a posteriori* prediction of dose from 7 days of treatment history, including 3 INR observations, was less than 10 seconds.

The dose prediction tool is based on a published population warfarin model for adults [21] that has been theoretically bridged to children through the use of physiological principles [22]. The model incorporates age, bodyweight, baseline and target INR, and *CYP2C9* and *VKORC1* genotype (defined or assumed) for *a priori* dose predictions, and uses doses and INRs from ongoing treatment for *a posteriori* dose revisions. The warfarin model was developed in NONMEM [23], which is the most commonly used software for non-linear mixed effects modeling of clinical PK and PD data. Dose optimization could in theory be performed using this software, but there are several reasons for moving to another environment. NONMEM, like other specialized software for non-linear mixed effects modeling, has i) a high knowledge threshold for use, ii) specific demands for data input, and iii) requires licensing of a program. All these aspects would impede the use of the model as a dose decision tool. An advantage of the tool compared with other warfarin dose algorithms, is that it can be used to adjust warfarin dosing *a posteriori* due to other known or unknown factors than those specifically included in the prediction model. When the *a posteriori* function is used, the tool will start by estimating individual model parameters based on the patient’s input data. The individual model parameters can be seen as the patient’s warfarin phenotype, and determines how the patient most likely will respond to therapy. When the individual model parameters are estimated all factors that affect the PK or PD of warfarin, e.g. regular exercise, vitamin K intake, interacting drugs or other medical conditions, will be taken into account and influence dose predictions. Another advantage of the tool is its ability to handle INR observations under non-steady-state conditions. INR observations that are measured during initiation of warfarin therapy or after dose changes give valuable information about an individual patient’s response to warfarin, both concerning rate and extent. The tool can use INR values from start of therapy and provide estimates of the expected INR at steady state. In theory, this means that patients can reach a stable maintenance dose in less time and with fewer dose adjustments and INR measurements than an empirical dosing regimen.

When comparing maintenance dose predictions from NONMEM and the Java based tool, there was a systematic difference with a bias (MPE) of -0.104 mg and an imprecision (RMPE) of 0.192 mg for the tool. These differences are relatively small and are not expected to influence dose recommendations when considering the limitations in available tablet strengths. That there is a difference between the tool and NONMEM may be explained by differences in i) the optimization algorithm used when estimating individual doses, and ii) the definition of target INR at steady state. The Java based tool defines the target INR as the mean INR during a dosing interval whereas NONMEM defines the target INR as the INR at 16 hours post dose.

## Conclusions

The predictive performance of the underlying published warfarin model has been extensively evaluated and shown to perform well in predicting the anticoagulant response in both children and adults [19,21,22,27]. The dosing tool needs to be evaluated prospectively before it can be recommended for use routinely in a clinical setting. However, even before a formal validation, it is possible to build confidence in the tool by using it for prediction of INR. Irrespective of whether the dose administered to a patient is derived from the tool or if it is an empirical dose, its accuracy can be evaluated by comparing predicted and observed INR values. A major limitation with the tool from a clinical perspective is that it has no save or printing function. However, there is at least one commercial dose-individualization software tool that have our warfarin models implemented, which has both a save and a printing function (www.doseme.com.au). It is important to emphasize that this type of decision support tool is not intended to substitute for the care by a licensed health care professional, such as a clinician, pharmacist or specialized nurse. It should rather be seen as a tool to help ensure efficient and consistent dose adjustment practices between prescribers and between different health care providers, irrespective of target INR or target population.

## Availability and requirements

**Project name:** Warfarin Dose Calculator 1.0.1

**Project home page:**www.warfarindoserevision.com

**Operating system(s):** Platform independent

**Programming language:** Java

**Other requirements:** Java Runtime Environment (JRE) 1.7.0 or newer

**License:** Apache Open source

**Any restrictions to use by non-academics:** No

## Additional files

Additional file 1:
**Warfarin Dose Calculator 1.0.1.** Provides important information on how to name data files for importation of treatment data into the Warfarin Dose Calculator. Additional file 2:
**Naming of data files.** Provides a template for the data required for importing a patient’s treatment history into the Warfarin Dose Calculator. Additional file 3:
**Format of treatment history.** Provides the Java-based decision support tool described in this paper.

## Abbreviations

A: drug amount in the body
CL: clearance
DR: dose rate
E_max_: maximum degree of inhibition
EDK_50_: dose rate resulting in 50% of maximum inhibition
INR: International Normalized Ratio
k_e_ or k10: first-order drug elimination rate constant
MPE: mean prediction error
MTT: mean transit time
PD: pharmacodynamics
PK: pharmacokinetics
RMPE: relative mean prediction error
V: volume of distribution
