# Supplementary material for: A Bayesian decision support tool for efficient dose individualization of warfarin in adults and children
Source: BMC Med Inform Decis Mak. 2015 Feb 7;15:7. doi: 10.1186/s12911-014-0128-0 (PMC4324411; doi:10.1186/s12911-014-0128-0)
Supplement: Additional file 2: — Naming of data files. Provides a template for the data required for importing a patient’s treatment history into the Warfarin Dose Calculator. [file 12911_2014_128_MOESM2_ESM.pdf]

Important information regarding naming of Excel-files for importation of treatment data into the Warfarin Dose Calculator

The file-name should consist of three parts, the first two separated by a hyphen. Example: AAAAA-XXXXXXXXXXZZZZ.

- The first part is flexible and can be a patients name, a code or something else. The number of characters is not critical.

- The second part must be the patients date of birth, written in the following format: yyyymmdd
  Example: 19640515 for a person born on May 15, 1964

- The third part must be numerical, with a combination of four digits. In Sweden these four digits when combined with the patient's data of birth forms a persons Social Security Number, which provides a unique identifier for each citizen.

Example of a valid file-name: Ann Smith-196405155208.xls
